# A Reply to “Comments on “A New Elliptical Model for Device-Free Localization””

**DOI:** 10.3390/s18030713

**Published:** 2018-02-27

**Authors:** Qian Lei, Haijian Zhang, Hong Sun, Linling Tang

**Affiliations:** 1 School of Electronic Information, Wuhan University, Wuhan 430072, China; leiqian_0521@whu.edu.cn (Q.L.); hongsun@whu.edu.cn (H.S.); 2 State Key Laboratory of Information Engineering in Surveying, Mapping and Remote Sensing, Wuhan University, Wuhan 430079, China; tanglinling@whu.edu.cn

**Keywords:** device-free localization, radio tomographic imaging, elliptical model

## Abstract

Recently, a comment paper on “A New Elliptical Model for Device-Free Localization” (Sensors 2016, 16, 577) has been presented, and the authors have provided a modified model. However, there are still some misunderstandings. In this reply, we further explain the proposed elliptical model in (Sensors 2016, 16, 577) to make it more understandable.

## 1. Introduction

**Figure 1:**
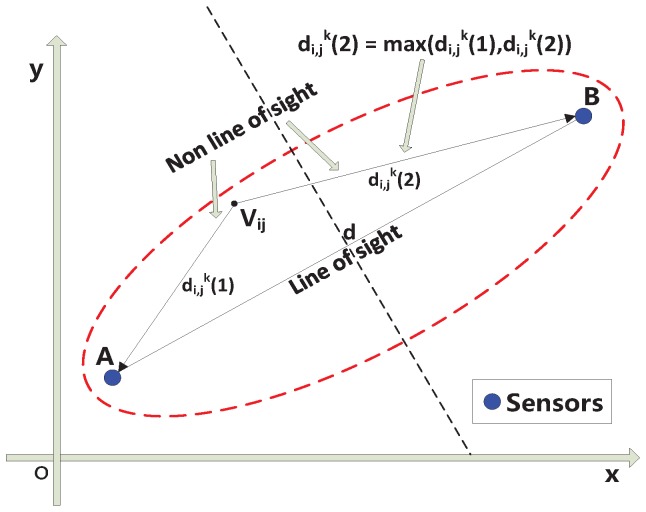
The communication paths inside one ellipse area.

To improve the localization accuracy, the elliptical model, which represents the communication link, is divided into line-of-sight (LOS) and non-line-of-sight (NLOS) paths [1]. The proposed elliptical model in [1] is illustrated in Figure 1, where *A* and *B* are sensors; *d* is the distance between *A* and *B*; $V_{ij}$ is the center of voxels in the ellipse area, *i* and *j* are the indices of voxels, $i=1,2,3...M_{1},j\;=\;1,2,3...M_{2},M_{1}\in N_{+},M_{2}\in N_{+}$; *k* is the index of links, which are expressed as ellipses in the experiment scenario; $d_{i,j}^{k}\left(1\right)$ is the distance between voxels *A* and $V_{ij}$, and $d_{i,j}^{k}\left(2\right)$ is the distance between *B* and $V_{ij}$. Based on the above elliptical model, we propose a new weighted formula as follows:

(1) $$W_{ij}^{k}=\left\{\begin{array}{c} \frac{1}{d}\left(k_{1}+max\left(d_{i,j}^{k}\left(1\right),d_{i,j}^{k}\left(2\right)\right)\right)\\ \;\;\;\;\mathrm{if}d_{i,j}^{k}\left(1\right)+d_{i,j}^{k}\left(2\right)<d+\lambda ,\;d_{i,j}^{k}\left(1\right)+d_{i,j}^{k}\left(2\right)\neq d\\ \frac{1}{d}\left(k_{2}+max\left(d_{i,j}^{k}\left(1\right),d_{i,j}^{k}\left(2\right)\right)\right)\\ \;\;\;\;\mathrm{if}d_{i,j}^{k}\left(1\right)+d_{i,j}^{k}\left(2\right)<d+\lambda ,\;d_{i,j}^{k}\left(1\right)+d_{i,j}^{k}\left(2\right)=d\\ 0\;\;\;\;\mathrm{otherwise} \end{array}\right.$$

where $k_{1}$ is a coefficient representing the obstacle to the communication on the NLOS path, and the value of $k_{1}$ is 2 by empirical experiments on the data provided by Prof. Neal Patwari [2]; $k_{2}$ is a coefficient representing the obstacle to the communication on the LOS path, whose value is 2.5 by empirical experiments on the data in [2].

## 2. Explanation and Analysis

### 2.1. Explanation of Our Formula

We would like to confirm that the proposed formula in (1) is reasonable to some extent and is not contrary to our description “*when a target stands on a line-of-sight (LOS) path, the influence on the communication link is greater than when a person stands on a non-line-of sight path inside the same weighting area*”. In our paper [1], we did not mention that “*the target’s influence on the link when a person stands near the LOS path is greater than that when a person is far from the LOS path*”. Instead, we did mention that “*when voxels were on non-line-of-sight paths, the less are the distances between voxels and the nearest sensors in the same link, the greater are the weightings values*”.

It should be emphasized that our proposed elliptical model in [1] has been developed based on the work in [2], and we use the same experiment scenario and data as in [2] to design the new elliptical model and the weighted formula in (1) in order to improve the accuracy of DFL. In [2], the experiment is conducted at the University of Utah, and a sensor network consisting of 28 sensors is deployed on a grassy area along the perimeter of a 21×21 foot square in an outdoor environment, where two trees are included. The experiment data in [2] are collected in this experiment scenario. Without considering the coefficients $k_{1}$ and $k_{2}$, the formula in (1) indeed cannot meet the description “the influence is greater when a person stands on the LOS path”. In order to ensure that the weightings of voxels on the LOS path are bigger than those of voxels on the NLOS path, the coefficients $k_{1}$ and $k_{2}$ are introduced. The procedure of determining the optimal $k_{1}$ and $k_{2}$ is elaborated in our paper [1], and we obtain the optimal values of $k_{1}=2$ and $k_{2}=2.5$.

**Figure 2:**
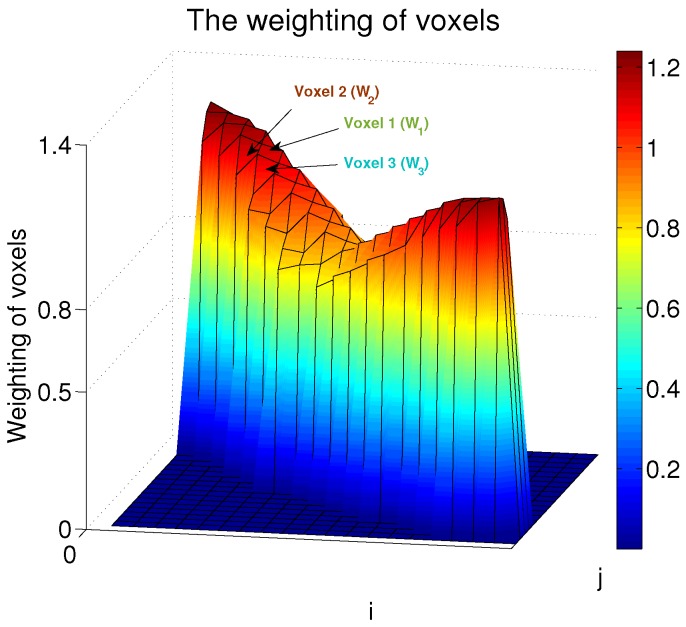
The weightings of voxels in one of the communication links.

In order to validate the rationality and validity of the proposed weighted formula (1) , the weightings of voxels in one of the communication links are computed based on the formula in (1) and the data in [2], and the results are depicted in Figure 2, where *Voxel* 1 is on the LOS path in the link, while *Voxels* 2 and 3 are on the NLOS paths. $W_{1}$, $W_{2}$, $W_{3}$ represent the weightings of the *Voxels*1,2,3, respectively. It is demonstrated that the values of $k_{1}$ and $k_{2}$ can always meet the description “*when a target stands on a line-of-sight (LOS) path, the influence on the communication link is greater than when a person stands on a non-line-of sight path inside the same weighting area*” and the description “*the less are the distances between voxels and the nearest sensors in the same link, the greater are the weightings values*”.

When we designed the weighted formula in (1) , we did not put the emphasis on the fact that “*the target’s influence on the link when a person stands near the LOS path is greater than that when a person is far from the LOS path*”, because the proposed weighted formula in (1) is specifically designed for the experiment environment in [2], where the number of voxels in the ellipse area is very limited, e.g., in Figure 2 there is only one NLOS path on both sides of the LOS path.

Finally, it should be explained that we indeed used the same scenario and data in [2], but when we transform the unit foot into the unit metre, we approximately write “6.3 m × 6.3 m” into “7 m × 7 m”, i.e., the experimental results in [1] are actually conducted based on the 6.3 m × 6.3 m scenario. Thanks for pointing out this problem, and we agree that when we write 7 m × 7 m (which actually should be 6.3 m × 6.3 m) in our paper, it is not rigorous. The authors in [2] provided the data to us, that is why we wrote “*this work is based on data provided by Neal Patwari, and the authors are grateful for his enthusiastic help*” in the Acknowledgments of our paper.

### 2.2. Analysis of the Modified Formula in the Comment Paper

In the comment paper, the authors propose a modified formula as below:

(2) $$W_{ij}^{k}=\left\{\begin{array}{c} \frac{1}{d}\left(k_{1}+\frac{1}{min(d_{ij}^{k}\left(1\right),d_{ij}^{k}\left(2\right))}\right)\\ \;\;\;\;\mathrm{if}d_{\mathrm{ij}}^{k}\left(1\right)+d_{\mathrm{ij}}^{k}\left(2\right)<d+\lambda ,\;d_{\mathrm{ij}}^{k}\left(1\right)+d_{\mathrm{ij}}^{k}\left(2\right)\neq d\\ \frac{1}{d}\left(k_{2}+\frac{1}{min(d_{ij}^{k}\left(1\right),d_{ij}^{k}\left(2\right))}\right)\\ \;\;\;\;\mathrm{if}d_{\mathrm{ij}}^{k}\left(1\right)+d_{\mathrm{ij}}^{k}\left(2\right)<d+\lambda ,\;d_{\mathrm{ij}}^{k}\left(1\right)+d_{\mathrm{ij}}^{k}\left(2\right)=d\\ 0\;\;\;\;\mathrm{otherwise} \end{array}\right.$$

furthermore, the authors also verify this formula by a real experiment. Firstly, we agree with that this modified formula meets the description “*the distance between a target and a LOS path is smaller, and the influence of the target is greater*”. However, how to obtain optimal values of the coefficients $k_{1}$ and $k_{2}$ is not introduced, which we think should be addressed. Most importantly, in order to prove the validity of a modified formula, it is necessary to simulate the final localization performance to this modified model, just like the works in [1,3,4,5]. The real experiment in the comment paper just proves that the modified formula in (2) satisfies the description “*the distance between a target and a LOS path is smaller, and the influence of the target is greater*”, and cannot prove the validity without conducting any simulation on localization accuracy. As a result, the authors in the comment paper could employ some measured data, e.g., the data in [2] to demonstrate the validity of the modified formula.

We think that the modified formula in (2) might be a more general model compared to the formula (1) , and can be applied in different experimental environments. If the authors in the comment paper are able to analyse the optimal selections of $k_{1}$ and $k_{2}$, and verify the modified formula on the accuracy of DFL by simulation, this kind of work will be meaningful.
